# A framework for improved collaboration on HTA in the Asia-Pacific region: a role for HTAsiaLink

**DOI:** 10.1017/S0266462324000588

**Published:** 2024-11-04

**Authors:** Ryan Rachmad Nugraha, Christian Suharlim, Rozar Prawiranegara, Arry Lesmana Putra, Mutia A. Sayekti, Armansyah Armansyah, Lusiana Siti Masytoh, Sweta Saxena, Anastasia Susanto, John C. Langerbrunner, Nurul Maretia Rahmayanti, Miyoung Choi, Budi Wiweko, Budi Hidayat

**Affiliations:** 1 Management Sciences for Health (MSH), Arlington, VA, Virginia; 2 Center for Health Financing and Policy, Ministry of Health Government of Indonesia, Jakarta, Indonesia; 3 United States Agency for International Development, Bureau for Asia/Technical Services, Washington, DC, USA; 4 United States Agency for International Development, Health Office, Jakarta, Indonesia; 5 National Evidence-Based Healthcare Collaborating Agency (NECA), South Korea; 6 Indonesian Health Technological Assessment Committee (InaHTAc), Jakarta, Indonesia; 7 Indonesia Medical Education Research Institute, Faculty of Medicine, Universitas Indonesia – Dr. Cipto Mangunkusumo General Hospital Jakarta, Jakarta, Indonesia; 8 Department of Health Policy and Administration, Faculty of Public Health, University of Indonesia, Depok, Indonesia

**Keywords:** HTAsiaLink, capacity building, knowledge exchange, health technology assessment, regional network

## Abstract

Countries frequently use health technology assessment (HTA) to set priorities for introducing new interventions or evaluating existing interventions; however, applying the tool effectively is heavily dependent on a country’s resources and capacity. Infrastructure and data, technical expertise, broad stakeholder involvement, and financial support are required to improve HTA processes. In the Asia-Pacific, HTAsiaLink was established to facilitate this practice, but strengthening and legitimizing this organization are needed to maximize its potential to support HTA institutionalization in the region. To realize this objective, HTAsiaLink can serve as a center of excellence while providing experiential learning and sharing information. As a learning hub, HTAsiaLink can share resources—particularly data—that can contribute to joint HTAs as done in the European Union and strengthen capacity in countries needing to develop their HTA expertise.

## Background

Countries working toward universal health coverage use health technology assessment (HTA) to provide the evidence they need for priority setting, however, their implementation proficiency varies. Contributing factors include infrastructure, such as a clinical data registry; organizational and staff capacity; and institutionalization of the HTA system (1,2). Countries need clinical data gathered during health service delivery to make better HTA decisions. Moreover, ample technical expertise among human resources improves the quantity and comprehensiveness of HTA evidence, and therefore, HTA output (3,4). HTA can be institutionalized in multiple ways, for example, one public agency is tasked to carry out assessments, or alternatively, one national agency does the appraisal, while agents such as academic researchers or think tanks carry out the assessment. Despite no “one-size-fits-all” process, countries with institutionalized HTA benefit from more efficiency and stability (5).

At the national level, HTA supplies needed health policy information, with support ranging from assessing a health intervention’s relative cost-effectiveness to include in a benefits package to disinvesting in health technologies (6). Additionally, HTA’s multiple stakeholders have different policy questions and needs; they comprise groups or individuals who can affect or who are affected by HTA decisions including industry; policymakers; clinicians and professional associations; and patients, families, and caregivers. HTA practitioners, who are typically health care professionals, epidemiologists, or health economists, are the experts who conduct HTAs, which are then appraised by stakeholders and/or HTA committees.

The variation in HTA output calls for harmonized efforts through regional collaboration that will spread productivity across countries. The World Health Organization has identified at least 10 regional networks that can provide support and share knowledge on HTA and the benefit package design process (7); in Asia-Pacific, HTAsiaLink plays this role. Through this paper, we explore ways to elevate HTAsiaLink in a way that enhances harmonized HTA collaboration in Asia-Pacific.

## HTA requirements and challenges

An effective HTA process requires resources such as research data and priority-setting frameworks as well as a strong foundation of (1,2):

- Regulation (rules)
- HTA literacy
- Transparency
- Skilled implementers
- Adequate funding
- Political commitment
- Institutionalization

Such complex interactions require an ecosystem that can support efficient yet high-quality HTA output, and these conditions are greatly affected by the HTA system’s maturity (8), which is often weak in low- and middle-income countries (LMICs). Constraints may be budgetary but may also come from a lack of research resources, external stakeholder involvement, or adequate internal ability to execute a high-quality HTA process such as using established study parameters to produce a national HTA report (1). For example, randomized controlled trial results are prioritized as clinical evidence, however, for LMICs with resource constraints, alternative methods are to synthesize study results from a literature search or use a real-world evidence approach. In either case, the ability of practitioners who perform assessments is key, but unfortunately often overlooked, which reduces assessment quality. Assessments and appraisals are frequently done in parallel, and both steps greatly depend on the number and diversity of experts available, which LMICs typically lack.

Robust data is crucial to HTA research, and better infrastructure in the form of cost databases and clinical registries for catastrophic diseases (e.g., cancer and cardiovascular diseases) yields higher-quality, more readily available information. Countries with well-established HTA infrastructure such as Australia and Japan have extensive secondary data sources and clinical registries that facilitate HTA analysis (9). To increase the amount of available data and evidence, the pharmaceutical industry could provide data for HTA practitioners to use in assessments, or the HTA agency could appraise industry results (10,11). However, even when these tools exist, the quality of data collection and analysis varies widely (12).

Other factors that can reduce a country’s HTA output are insufficient political prioritization and poor institutionalization, which leads to inadequate management, communication, and coordination to support the generation and use of HTA studies (13,14).

## Meeting demand for information-sharing: HTAsiaLink

Demand for sharing information within Asia led to the establishment of HTAsiaLink, a collaborative network of HTA agencies. Initiated in 2011, HTAsiaLink’s mission is to encourage the use of HTA evidence for universal health coverage by providing a platform for catalyzing HTA diffusion into countries’ health systems (15). Currently, HTAsiaLink hosts 44 agencies as organizational members from 17 different countries in Asia-Pacific, including Australia.

Annually, HTAsiaLink holds a conference where practitioners from the network members (and often external organizations) share updates on HTA-related information. What is unique is that HTAsiaLink member countries commit their own domestic resources (with assistance from external parties) to organize this scientific exchange. Such cohesiveness has made HTAsiaLink a distinctive community of public HTA practitioners. In addition, to meet growing HTA demand, other regional efforts are seeking to harmonize HTA practice; for example, the Association of Southeast Asian Nations (ASEAN) Secretariat is working to harmonize HTA among its member states (16), and the real-world data in Asia for Health Technology Assessment in Reimbursement (REALISE) consortium is streamlining the use of real-world evidence (17). These joint efforts are in the early stage of development.

## Leveraging HTAsiaLink’s potential as a regional knowledge hub

As noted, the quantity and quality of countries’ resources affect their ability to produce HTA results, so *resource-sharing* can help those with more limited resources make more timely policy decisions. HTAsiaLink could encourage their mature HTA agency members to share their analysis tools so that HTA results can be replicated. In this way, agencies with fewer resources can analyze new data using previously published methods, ensuring that the results are still reliable (18). To boost the HTA output needed to meet demand, countries can use decisions arising from similar situations (or *results-sharing*), and HTAsiaLink could manage a knowledge and collaborator hub to simplify information-sharing on novel HTA evidence. HTAsiaLink could also support *data-sharing*, which is another form of regional collaboration. Data routinely collected during health care delivery can generate real-world evidence that countries with insufficient primary data and evidence, particularly from trials and cost-effectiveness studies, can use in HTA decision-making (19). For example, countries with updated registries for a disease such as cancer could share that data with similar countries that lack a cancer registry but need HTA evidence. Despite the potential benefit, applying data-sharing to policy decision-making has prerequisites and entails data-sharing agreements and policymakers in the region would need to reach a consensus on how to share sensitive information. In addition, transferability may pose a concern, particularly on how to adapt foreign data that might not fully apply to the local population. Adjusting local evidence to the shared data to use as a supplement, as well as creating a collaboration framework for data governance may alleviate data-sharing issues and facilitate collaboration (20,21). Caution should be taken, however, because generally, methods and tools to accurately transfer real-world cost-effectiveness estimates are lacking.

Since its launch in 2011, the HTAsiaLink annual conference has evolved as a platform where members share information on HTA findings for priority-setting and technical know-how including tools, methodologies, and results. To increase its usefulness and sustainability, HTAsiaLink can consider the following ideas to become a more robust learning hub and expand its knowledge-sharing methods:

- *Center for excellence.* By establishing itself as a center for excellence, HTAsiaLink would need to further develop its management of knowledge and the ability to share it with other stakeholders (22). A center for excellence could maximize the experience and expertise of those whose practices are at the forefront of HTA, particularly from high-income countries with an established procurement system as part of a public-sector health care system.
- *Experiential learning.* Experiential learning or learning by doing, helps boost implementers’ aptitude in an area while strengthening research partnerships (23). This type of reciprocal knowledge exchange is useful when countries would like to learn more about HTA but lack a process to conduct their own HTA studies. Countries can also use experiential learning to train their researchers in HTA analysis and increase HTA output. Experiential learning focuses on joint HTA research between more- and less-experienced countries, where they can apply resource-sharing and data-sharing techniques. In 2014, Thailand’s Health Intervention and Technology Assessment Program (HITAP) used experiential learning to help Indonesia conduct a local economic evaluation assessment for drugs in its Package of Essential Medicines for Noncommunicable Diseases (24). HITAP shared its expertise while Indonesia conducted the research using its own data and held discussions with national stakeholders.
- *Optimizing existing networks for information-sharing.* Other collaborative HTA networks, such as the European Network for Health Technology Assessment (EUnetHTA) and the International Network of Agencies for Health Technology Assessment (INAHTA), provide opportunities for their members to share best practices and design joint activities. Since 2022, EUnetHTA has promoted the use of HTA and the reduction of redundancies through voluntary joint collaboration across Europe (25). The INAHTA data repository serves a similar purpose by providing public access to updated evidence from ongoing and published HTA reports including reviews, guidelines, protocols, journals, and research articles (27); similarly, the INAHTA and Health Technology Assessment International collaborated on creating the HTA Glossary (htaglossary.com), which is a publicly available dictionary of HTA terms. This is in addition to the European Union’s 2021 law that requires member states to develop a joint process for clinical assessment that will be integrated into national HTA reports (28).

**Figure 1.**
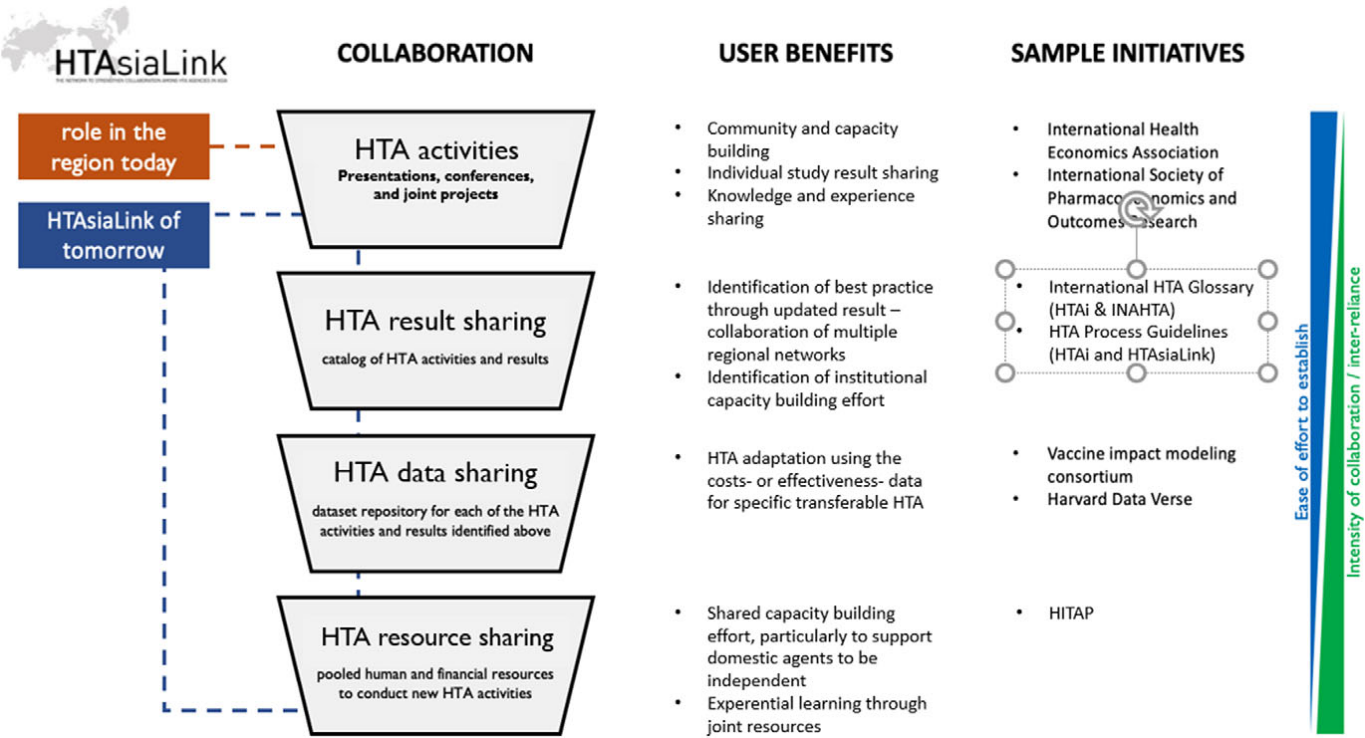
HTAsiaLink’s potential as a multi-purpose HTA resource for the Asia-Pacific region.

As a learning hub, HTAsiaLink can share resources, particularly data, and build countries’ policymaking capability (Figure 1). For example, HTAsiaLink members could share datasets to facilitate joint research, or they could exchange researchers to support learning from a more mature agency or a less mature agency to carry out its own HTA study. Neighboring countries with similar demographic or epidemiologic challenges may also benefit from adapting others’ policy decisions, as feasible. One example is ThinkWell’s Immunization Delivery Cost Catalogue, which combines countries’ vaccination program cost data into one single pool of evidence (28). Fragmented health financing and not prioritizing human resource improvement often lead to inadequate financing for increasing HTA ability (29). A collaborative hub such as HTAsiaLink could facilitate the information or even share resources in creating shared resources altogether utilizing each member’s country experts. For example, in 2024, the regional network, HTAi made Memorandum of Understanding (MoU) with (1) INAHTA to create HTA Glossary which may serve as HTA’s first go-to terminology and go-to reference, and (2) HTAsiaLink to create HTA process guidelines (30). Moreover, HTAsiaLink could also promote institutionalization, which would increase domestic financial resources for building HTA capacity.

When sharing data, countries need to consider the level of effort and intensity of the collaboration (the green and blue upward-downward arrow in Figure 1). To achieve the desired level of collaboration, a phased approach should be considered by sharing results and tools in the short term and sharing resources in the longer term. Where HTA is a competing priority among other policy initiatives, the country can adjust its approach based on resource allocation.

Multi-stakeholder partnership also needs to be reinforced, such as between countries and external partners, particularly to sustain HTA efforts. A development aid mechanism, for example, can be used to strengthen collaborative platforms such as HTAsiaLink; in addition, effective aid can strengthen priority-setting capability and process as countries invest in their HTA ecosystems and use the tools to increase the value of their health spending. This is a marginal aid approach, where domestic financing is allocated to the core package of the highest-priority, most cost-effective health services, and products, while external financing is used to fund the next most cost-effective service, which eases the long-term transition to full domestic financing (31). HTAsiaLink can use its convening power to promote collaboration among LMIC leaders, academics and researchers, global health donors, and multilateral organizations to advocate for and facilitate a marginal aid approach to priority-setting. To increase transparency, development partners can evaluate the use of outcome-based aid meant to support HTA efforts.

## Conclusion

To increase the maturity of HTA systems throughout Asia-Pacific, HTAsiaLink can provide a solid collaborating platform for countries to boost their HTA capacity and better meet the demands for HTA development. HTAsiaLink can also encourage and facilitate knowledge- and resource-sharing to supply policymaking evidence, particularly in the adoption of new health technologies. In addition, HTAsiaLink should serve as an external as well as internal hub by engaging with other regional networks and similar platforms to share experiences. Finally, innovative ways to support network development, including external financing through a marginal-aid mechanism, will increase countries’ domestic HTA capability.
